# Diversity of ESBL-producing *E. coli* in various water and sediment types

**DOI:** 10.1371/journal.pone.0338703

**Published:** 2025-12-09

**Authors:** Leon Maric, Maja Rupnik, Sandra Janezic

**Affiliations:** 1 Department for Microbiological Research, Centre for Medical Microbiology, National Laboratory of Health, Environment and Food, Maribor, Slovenia; 2 Biotechnical Faculty, University of Ljubljana, Ljubljana, Slovenia; 3 Faculty of Medicine, University of Maribor, Maribor, Slovenia; National Research and Innovation Agency, INDONESIA

## Abstract

Antimicrobial resistance is a major global health threat, and aquatic ecosystems serve as critical interfaces between human activity and the environment, providing reservoirs and pathways for the spread of resistant bacteria. In this study, we investigated the prevalence and diversity of ESBL-producing *Escherichia coli* across multiple aquatic environments in Slovenia by analysing water, sediment, and wastewater treatment plant (WWTP) influent samples collected between June 2023 and April 2024. ESBL-producing *E. coli* was detected in 93% of WWTP influents, 61% of water, and 35% of sediment samples. From 315 positive samples, 564 isolates were subjected to whole genome sequencing. In total, 134 MLST-sequence types (STs) were identified, with globally prevalent lineages (ST131, ST38, ST10, ST69, ST1193) occurring across regions and sample types. Clonally related isolates occurred in geographically distant sites, and persistent strains were observed over time, suggesting both local persistence and broader dissemination. Fourteen distinct ESBL genes were detected, predominantly plasmid-encoded *bla*_CTX-M-15_. Water samples captured the greatest ST diversity, whereas sediment and WWTP influents provided complementary insights. Combining sample types improved detection of overall diversity, with water and sediment or WWTP samples providing the most comprehensive overview. Our findings demonstrate widespread and dynamic dissemination of ESBL-producing *E. coli* in Slovenian aquatic environments and emphasize the value of integrated sampling strategies for effective surveillance.

## Introduction

The environment is increasingly recognized as an important component of the One Health framework, particularly due to its role as both a reservoir and a route for the dissemination of antimicrobial resistant (AMR) bacteria [1,2]. The environment also plays an important role in the overall ecology of one of the most concerning AMR bacteria; extended-spectrum beta-lactamase (ESBL)-producing *Escherichia coli* [3]. The widespread occurrence of ESBL-producing *E. coli* in the environment is largely attributable to wastewater effluents/pollution and other anthropogenic activities. Further spread between aquatic ecosystems can occur through wildlife, ultimately posing a significant exposure risk to humans [4,5].

Most research on ESBL-producing *E. coli* in the environment is focused on river surface waters and wastewater treatment plant (WWTP) samples, while other aquatic environments, such as lakes, gravel pits, or ponds, remain unexplored [6,7]. Studies focusing on ESBL-producing *E. coli* in rivers typically compare water samples from upstream and downstream sites of the city or WWTP effluent discharge. Downstream sites reflect inputs from urban communities, such as sewage and wastewater, whereas upstream sites may receive some human and animal influents but mainly serve as reference points to assess the impact of urban sources downstream [8].

WWTP influent and effluent and river water samples are the most commonly used metrics to study the prevalence and diversity of ESBL-producing *E. coli* in the environment. However, it remains unclear which sample type (or a combination of sample types) provides the most comprehensive information on the prevalence and diversity of ESBL-producing *E. coli*. Sampling WWTPs does not require ethical approval; however, obtaining samples often requires formal permission [9]. In contrast, sampling water or sediments from rivers and other aquatic environments involves fewer, if any, administrative barriers. Sediments, though often overlooked, are increasingly recognized as important reservoirs of antibiotic resistance and may also be relevant for such assessments [10–12].

The aim of this study was to: (i) expand our knowledge on the prevalence and diversity of ESBL-producing *E. coli* across various aquatic environments and different sample types in Slovenia; (ii) assess the prevalence and diversity of clonal strains; and (iii) compare sample types (water, sediment, WWTP) to determine which better represent the overall environmental diversity of ESBL-producing *E. coli*.

## Materials and methods

### Water, sediment, and WWTP influent sampling

Water and sediment samples were collected every two months from June 2023 to April 2024 across different freshwater aquatic environments in three geographically distinct regions: region A, region B, and region C (**S1 Fig**, **Table 1 in** **S1 File**). All sites were sampled six times, except for the WWTP influent sites, which were sampled five times. Environmental sampling complied with Slovenian national legislation and institutional guidelines. No specific permits were required, as sampling was performed non-destructively at publicly accessible river sites and with permission from wastewater treatment plant operators. Detailed information about all 28 sampling sites across aquatic environments and the number of collected samples per each is provided in Table 1 in S1 File.

**Table 1:** List of the ten most common MST groups with clonal isolates collected across different regions, sample types, and months.

MST	ST	No. of isolates	No. of samples	Region	Sample type	Month
MST_1	2179	15	14	A, B	Sediment, water, WWTP	June/July, Aug, Oct, Dec, Feb, Apr
MST_2	12548	12	7	B	Sediment, water	June/July, Aug
MST_3	1193	11	8	A, B	Sediment, water, WWTP	June/July, Oct, Dec, Apr
MST_4	16433	11	11	A	Sediment, water, WWTP	June/July, Oct, Dec, Feb, Apr
MST_5	540	9	6	A, C	Sediment, water, WWTP	Aug, Oct, Dec, Feb
MST_6	44	9	4	A	Sediment, water	Aug
MST_7	636	7	7	A, B, C	Sediment, water, WWTP	June/July, Aug, Dec, Feb
MST_8	5846	7	4	C	Sediment, water	June/July, Aug
MST_9	38	6	5	A, C	Sediment, water, WWTP	Aug, Dec, Feb
MST_10	10	6	5	A, B, C	Water, WWTP	June/July, Aug, Dec, Apr

The sampled aquatic environments included waterbodies with different hydrological characteristics, such as rivers (11 sampling sites on three different rivers), tributaries (n = 4), artificial lakes (reservoir lakes located at rivers included in sampling) (n = 4), a natural lake (n = 1), gravel pits (n = 4), and a pond (n = 1). Additionally, WWTP influents (n = 3; one WWTP in each region) were sampled. In each region, the main river (Ledava in region A, Drava in region B, and Sava in region C) was sampled at the nearest accessible site upstream and downstream of WWTPs effluent discharge points. Due to intensive agricultural activity in region A, an additional site in the agricultural area along the river Ledava was sampled. Tributaries with potential influences from biogas plants, farms, and landfills, and the tributary with a history of pollution incidents, were also included (n = 4). Reservoir lakes (n = 4) included in the study were either integrated in all three sampled rivers (n = 3) or selected due to previously detected antibiotic residues (n = 1). An additional natural lake, known for human activities such as swimming and bathing, was also included. Sampled gravel pits selected for this study have been transformed into natural systems used for recreational purposes like fishing, bathing, and swimming.

Water and sediment samples were collected at a depth of 20–30 cm whenever possible. In cases where the water was too shallow, samples were collected at the maximum available depth. At each site, 0.5 L of water and at least 25 g of sediment were collected in sterile bottles and 50 mL centrifuge tubes, respectively. For the WWTP influent, 0.5 L of water was collected as 24-hour flow-proportional composite samples. Samples were stored at 4°C and processed in the laboratory within 24 hours of sampling [13].

### Isolation of ESBL-producing *E. coli*

Isolation of ESBL-producing *E. coli* from sediment, water, and WWTP influent samples was conducted according to the WHO guidelines [8] and methodology described previously [13]. Briefly, 200 mL of water was filtered through 0.45 µm filtration papers (WhatmanTM, Germany), which were cultivated on Tryptone Bile X-glucuronide agar (TBX) (Biolife, Italy) with 4 µg/mL cefotaxime sodium salt (CTX) (Sigma-Aldrich, USA). Sediment (25 g) was mixed with sterile water to a final volume of 50 mL, sonicated, and then 10 mL of the supernatant was subjected to filtration and cultivation on TBX with CTX. For water and sediment samples, up to five colonies were collected from each plate. WWTP samples (100 µL) were directly inoculated onto TBX agar plates with 4 µg/mL CTX, and up to 20 presumptive ESBL-producing *E. coli* colonies were collected. For all samples, species identification was performed by matrix-assisted laser desorption ionization time of flight mass spectrometry (MALDI-TOF) (Bruker, Germany). ESBL production was confirmed by the disk diffusion method according to EUCAST guidelines (Eucast: Clinical Breakpoints and Dosing of Antibiotics, 2025) using ceftazidime + cloxacillin (CAC; 30 µg) and cefotaxime + cloxacillin (CTC; 30 µg) disks and their combinations with clavulanic acid (CALC or CTLC; 30 µg/10 µg) (Liofilchem, Italy). ERIC-PCR (enterobacterial repetitive intergenic consensus PCR) was performed on all isolates from each sample to identify and exclude potential clones [14]. Isolates were stored at – 80°C.

### Whole genome sequencing

For whole genome sequencing (WGS), genomic DNA was extracted using the QIAamp DNA **Mini Kit (Qiagen, Germany) according to the manufacturer’s protocol for Gram-negative** bacteria. Libraries were prepared using the NEBNext Ultra II FS DNA Library Prep Kit for Illumina (New England Biolabs, UK). Sequencing was performed on the Illumina NextSeq 2000 platform (Illumina, USA), generating paired-end raw reads with either 2 × 150 bp or 2 × 300 bp read lengths. The short-read sequencing data were deposited in the European Nucleotide Archive (ENA) under the project numbers PRJEB87212 (region A) and PRJEB90475 (regions B and C). ENA accession numbers for each isolate are listed in **Table 7 in** **S1 File**.

### Genomic analysis

After sequence quality control, *de novo* assembly of trimmed reads was performed using SPAdes (v3.13.0) using the *--careful* option, while all other parameters were kept at their default settings [15]. Multilocus sequence typing (MLST) [16] and core-genome MLST (cgMLST) were conducted in Ridom SeqSphere+ (v10.5.1) [17], applying a maximum allelic difference threshold of 10 alleles to define MST clonal groups. An allele-based neighbor-joining (NJ) phylogenetic tree was generated from the cgMLST data, also within SeqSphere + . Antimicrobial resistance gene detection and plasmid prediction were performed using AMRFinderPlus (v3.11.26; database version 3.11) [18] and MOB-suite (v3.1.8) [19], respectively. MOB-suite’s plasmid primary clusters were defined as a group of similar plasmids based on MASH distances, using a clustering threshold of 0.06. Phylogroup determination was conducted with EzClermont (v0.7.0) (https://github.com/nickp60/EzClermont). Data was visualized using R (v4.4.1) [20] and iTOL [21]. The map of Slovenia, showing rivers and sampling sites, was generated in RStudio using data from the national Open Data portal of Slovenia (https://podatki.gov.si/pogoji-uporabe#). Following packages were used: sf [22], rnaturalearth [23], rnaturalearthdata [24], osmdata [25], and ggspatial [26]. Sampling points were added approximately for schematic purposes.

### Statistical analysis

Statistical analyses were performed using R version 4.4.1 [20]. MLST sequence types (ST) and phylogroups diversity was measured as Richness or Shannon index using vegan v2.6.8 [27]. Differences in ST diversity across sources were tested with ANOVA and Student’s *t*-test with Benjamini-Hochberg (BH) correction for multiple comparisons were performed using rstatix v0.7.2 [28]. Additionally, the composition of STs and phylogroups was assessed using PERMANOVA based on Jaccard dissimilarity, with following packages: vegan v2.6.8 [27], rstatix v0.7.2 [28], RVAideMemoire v0.9.83.7 [29], EcolUtils v 0.1 [30]. Homogeneity of multivariate dispersion (β-dispersion) among groups was tested using the betadisper function from vegan v2.6.8 [27]. To compare the proportions of detected STs, ESBL genes, and primary plasmid clusters between sample types, a one-sided z-test of differences in proportion was performed, with results expressed as p-values. A significance level of 0.05 was applied for all statistical tests.

## Results

In total, 315 samples were collected: 15 water samples from WWTP influents, 150 water and 150 sediment samples from various water bodies. ESBL-producing *E. coli* was detected in 14 (93%) WWTP influent samples, 92 (61%) freshwater samples, and 53 (35%) sediment samples (**Tables 2-3 in** **S1 File**).

WWTP influents showed the highest detection rates, with 93% of samples testing positive. Among aquatic environments, all river/water samples were positive (upstream, downstream, and in agricultural area). River sediment samples also showed relatively high positivity rates: 50% upstream, 69% downstream, and 83% at the agricultural site. Tributary sites showed moderate detection rates, with 38% of water samples and 29% of sediment samples testing positive. Reservoir lakes and gravel pit sites had low sediment positivity (8%) and moderate-to-low water positivity (54% and 13%, respectively). Pond samples showed low positivity, with 17% positive water samples and no positive sediment samples. Lake samples tested negative in both sample types.

Although this study was not specifically designed to quantify concentrations of ESBL-producing *E. coli*, colony-forming unit (CFU) estimates for presumptive ESBL-producing *E. coli* are provided based on countable plates (**Table 2 in** **S1 File**, **S2 Fig**).

Following the collection of up to five or 20 colonies per sample, species identification, confirmation of ESBL production, and selection of unique ERIC-PCR profiles, a total of 564 isolates were selected for the detailed WGS analysis: 128 from sediment samples, 284 from water samples, and 152 from WWTP influents **(**S3 Fig**)**. Strains originating from river sediments were further divided into 29 river/upstream and 58 river/downstream of WWTP, 19 from agricultural river site. Strains originating from river water samples were also from river/upstream (n = 77), river/downstream (n = 128) and river/agricultural site (n = 18). An additional 83 isolates were from tributaries (24 water, 16 sediment), reservoirs (32 water, 4 sediment), gravel pits (n = 6), and a pond (n = 1). Metadata for all collected isolates are provided in **Table 4 in** **S1 File**.

### Diversity of ESBL-producing *E. coli* in water, sediment, and WWTP

**Fig 1:**
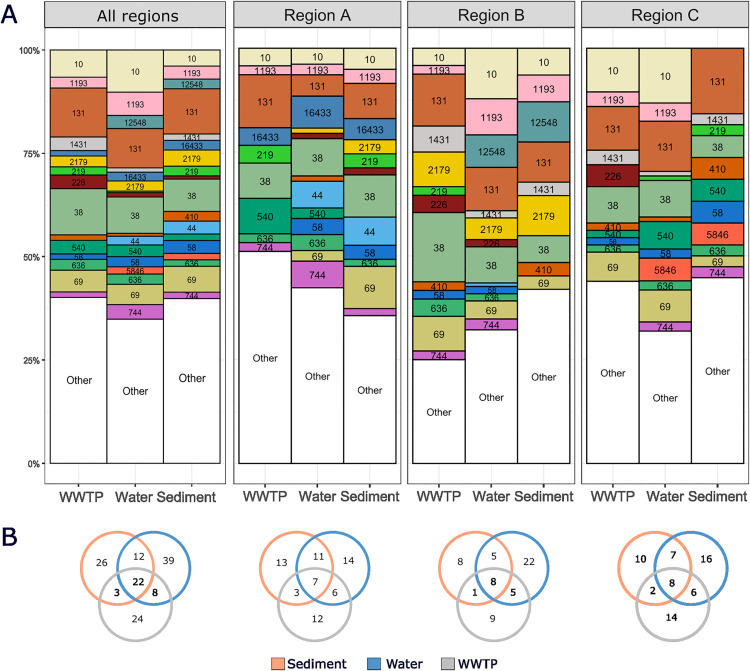
Comparison of prevalence of ESBL-producing *E. coli* across WWTP, water, and sediment samples. **(A)** Relative abundance of the most common STs detected in WWTP, water, and sediment for all regions combined and for each region separately. STs occurring fewer than four times are grouped under “Other.” Each ST is labeled and color-coded according to its identity. **(B)** Venn diagrams illustrating the distribution of STs in all regions combined and for each region separately. Each circle is colored according to the legend below.

A total of 134 different MLST-STs were found among all 564 isolates (**Fig 1A, Table 1 in** **S2 File**). Nine novel STs were identified, one found in sediment, five in water, two in WWTP, and one in both sediment and WWTP. Among 128 sediment, 284 water, and 152 WWTP isolates, 63, 81, and 57 different STs were identified, respectively. Twenty-two (16%) STs were found in all three sample types (sediment, water, and WWTP samples), 39 STs (29%) were found only in water, 26 STs (19%) only in sediment, and 24 (18%) only in WWTP samples (**Fig 1B**). ST diversity differed significantly between water and sediment samples and between water and WWTP samples, while no significant difference in ST diversity was found between sediment and WWTP samples (ANOVA, F = 7.024, p = 0.00772) (**Table 5.1 in** **S1 File**).

When comparing the distribution of STs across all three regions, 18 (13%) STs were found in all three regions, while many were region specific: 34 (25%) STs were found only in region A, 30 (22%) only in region B, and 35 (26%) only in region C (**Tables 2-3 in** **S2 File**). It is important to note that a large proportion of these region-specific STs (n = 75/99, 76%) were singletons. The most prevalent STs that were found in all regions and sample types were: ST131 (n = 59; 10.5%), ST38 (n = 52; 9.2%), ST10 (n = 44; 7.8%), ST69 (n = 30; 5.3%), and ST1193 (n = 24; 4.3%). Prevalent STs that were region-specific included ST16433 (11 isolates; 11%) found only in region A, ST12548 (12 isolates; 12%) in region B, and ST5846 (7 isolates; 7%) in region C. Statistical analyses revealed no significant differences in ST diversity among regions (ANOVA, F = 0.076, p = 0.927). However, the composition of STs differed significantly between regions (PERMANOVA, R² = 0.175, F = 1.5877, Pr(>F) = 0.001) (**Table 5.2 in** **S1 File**).

**Fig 2:**
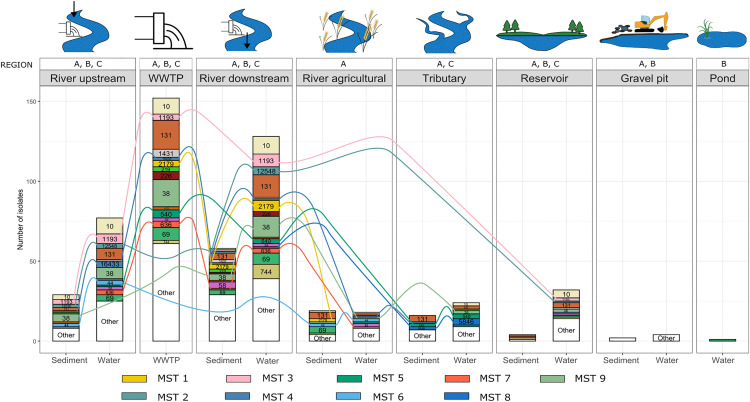
ST prevalence and clonal spread across aquatic environments and WWTP influents. The figure illustrates the distribution of STs and most prevalent clonal groups (MST) in sediment and water samples of different aquatic environments, and WWTP influents. Each ST is labeled and color-coded according to its identity. Lines are color-coded according to ST and connect clonal isolates. Only the nine most prevalent clonal clusters are shown. Labels above the charts **(A, B,** and **C)** indicate the regions where each aquatic environment was sampled.

ST diversity was also examined between different aquatic environments and WWTP samples (**Fig 2, Table 4 in** **S2 File**). Among all detected STs, ST131 showed the widest distribution, being present in six different aquatic environments (all except the gravel pits) and in WWTP. Other widely distributed STs included ST38, ST10, and ST69, all found in five aquatic environments (all except gravel pits and a pond) as well as in WWTP samples. ST diversity differed significantly among some aquatic environments (ANOVA, F = 32.72, p = 2.34E-12), indicating that certain environments harbour distinct ST compositions (**Table 5.3 in** **S1 File**). No significant differences in ST diversity were observed among gravel pits, reservoirs, agricultural river area, or tributaries. In contrast, WWTP samples showed significantly different ST diversity compared to all other sites, except for the river site downstream of the WWTP. Significant differences in ST diversity were also observed between the river site upstream of WWTP effluents and all other aquatic environments (**Table 5.3 in** **S1 File**). The distribution of STs was also examined over time, across each sampling point in each region; however, no clear seasonal patterns were observed (**S4 Fig****, Tables 5-6 in** **S2 File**).

**Fig 3:**
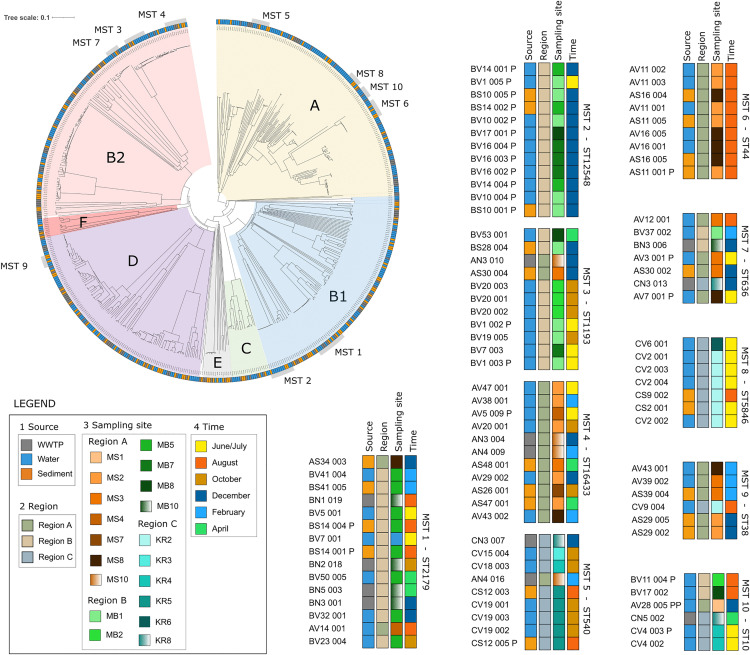
Phylogroups and ten most prevalent MST clonal groups. The figure shows a neighbor-joining phylogenetic tree with identified phylogroups, annotated by sample type: sediment (orange), water (blue) and WWTP (grey). Ten largest MST clonal groups are labeled. Additionally, heatmaps for each clonal group illustrate variation in source types, regions, sampling sites, and sampling time.

The most common phylogroups identified were A (27%), B1 (20%), B2 (24%), and D (20%) (**Fig 3, Table 6 in** **S1 File**). Phylogroups were consistently prevalent across all sample types, without a clear predominance in either sediment, water, or WWTP. Statistical analyses indicated no significant variation in phylogroup diversity (ANOVA, F = 1.215, p = 0.326) or composition (PERMANOVA, R² = 0.148, F = 1.2148, Pr(>F) = 0.291) among sample types (**Table 5.4 in** **S1 File**). In contrast, regional differences were detected in diversity based on the Richness index (ANOVA, F = 4.672, p = 0.027), although not when using the Shannon index, which incorporates relative abundances (ANOVA, F = 1.514, p = 0.252) (**Table 5.5 in** **S1 File**). No significant differences in phylogroup composition were observed between regions (PERMANOVA, R² = 0.1711, F = 1.5482, p = 0.208).

### Clonal strains were found in geographically distant environments

Out of 564 isolates, 287 (51%) grouped into 89 distinct MST clonal groups, while the remaining 277 were singletons (**Table 1 in** **S3 File**). Out of 89 MST groups, 10 groups included isolates from all three sample types, i.e., sediment, water, and WWTPs (three to 15 isolates per MST group). Eighteen MST groups included isolates from sediment and water samples, six groups from sediment and WWTP samples, and 14 groups from water and WWTP samples. The remaining 41 clonal groups contained isolates from only a single sample type. The ten most common MST clonal groups are presented in **Table 1** and **Fig 3**.

MST clonal groups were compared across regions, aquatic environments, time points, and locations (**Figs 2-3**, **S3 File**). Only three clonal groups (MST 7, MST 10, MST 39) included isolates from all three regions, while most were restricted to one or two regions (**Table 2 in** **S3 File**). Approximately half of the clonal groups (n = 48, 54%) included isolates that were shared by at least two aquatic environments, with MST 4 showing the widest distribution across five environments (**Table 3 in** **S3 File**). Most clonal groups included isolates from WWTP samples and the downstream river site (n = 21) (**Table 2**). No clonal groups containing isolates from gravel pits and other aquatic environments were identified.

**Table 2:** Matrix of MST groups indicating the number of MST groups with isolates from each pair of sample types.

	(1)	(2)	(3)	(4)	(5)	(6)	(7)
(1) River upstream							
(2) River downstream	10						
(3) River agricultural	5	13					
(4) Tributary	4	4	2				
(5) Reservoir	6	6	0	0			
(6) Gravel pit	0	0	0	0	0		
(7) Pond	0	1	0	0	0	0	
(8) WWTP	8	21	10	2	6	0	1

Temporally, isolates from 48 clonal groups were detected at multiple sampling points, while 41 were detected only once. For example, the largest clonal group, MST 1, included isolates present throughout the year, whereas MST 6 included isolates detected only in August. (**Table 1**). Detailed information about the prevalence of MST clonal groups through time is presented in **S5 Fig** and **Table 4 in** **S3 File**.

Spatially, most clonal groups (n = 69, 78%) contained isolates that were limited to one or two sampling sites, though some (n = 20, 22%) contained isolates that were more widespread, with MST 3 (ST1193; n = 6 sampling sites) and MST 4 (ST16433; n = 6 sampling sites) being the most prevalent across sites (**Table 5-8 in** **S3 File**).

**Fig 4:**
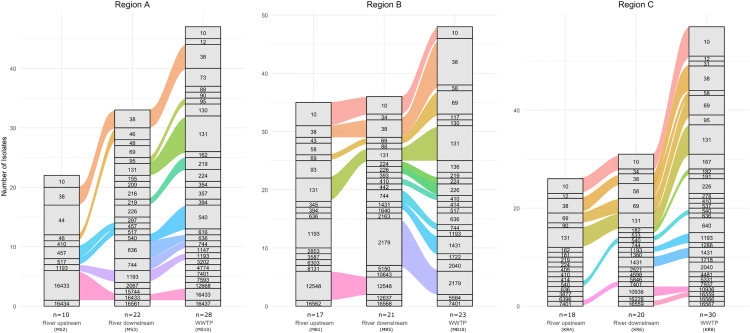
ST diversity and distribution in river sites upstream and downstream of WWTP effluent, and in WWTP influents. The figure illustrates the distribution of STs in river sites upstream and downstream of WWTP effluents, as well as in WWTP influent samples, across three different regions. Shared STs between upstream and downstream sites and between downstream site and WWTP influent, are connected by lines, indicating the potential contribution of STs from upstream site or WWTP to downstream site. Each line color represents a distinct ST. Numbers below the bars indicate the number of distinct STs identified at each sampling site. Sampling locations are indicated below each sampling site.

In each region, one upstream and one downstream river site relative to the WWTP effluent were selected along each river. Downstream sites were compared with upstream sites and WWTP influent to assess the potential contribution of WWTPs (**Fig 4**, **S6 Fig**). Across all regions, WWTP samples exhibited the highest diversity of STs, followed by downstream and upstream sites. More STs and MST clonal groups were shared between WWTP influent and downstream sites than between upstream and downstream river sites, suggesting that WWTP effluents contribute to the ST composition observed at downstream river sites. Certain clonal groups, such as MST 1 (C1_2179 on Fig 6 in S1 Figs) in region B, persisted throughout the year and were consistently detected in WWTP influent and downstream river sites, indicating continuous dissemination.

**Fig 5:**
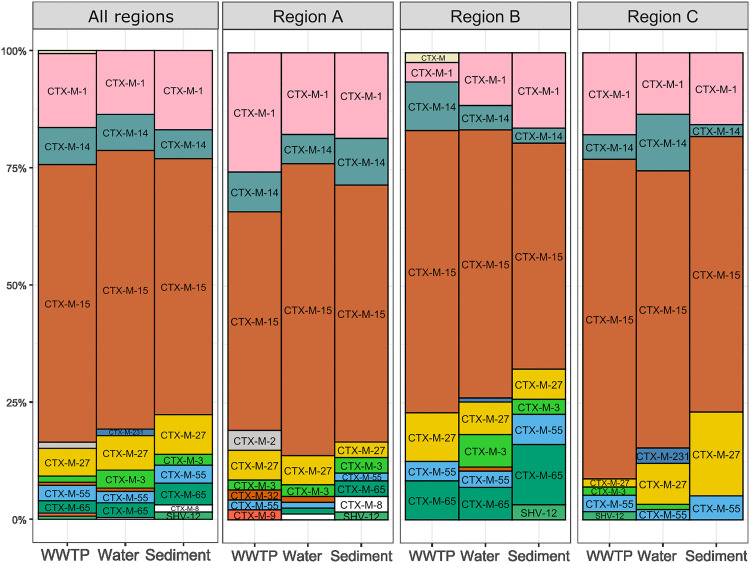
Comparison of ESBL genes prevalence in WWTP, water, and sediment. Relative abundance of ESBL genes detected in WWTP, water, and sediment samples for all regions combined and for each region separately. Each ESBL gene is labeled and color-coded according to its identity.

**Fig 6:**
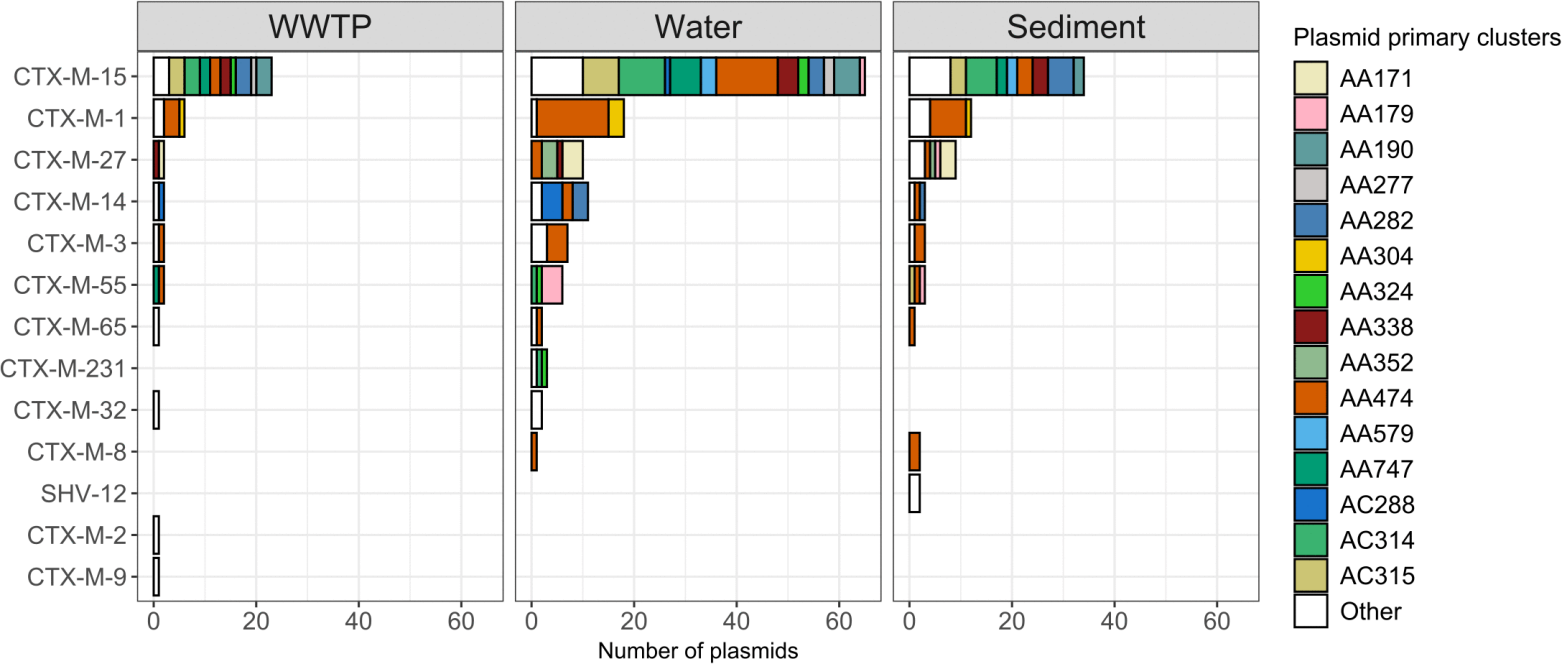
Distribution of ESBL genes in *E. coli* across different MOBSuite’s plasmid primary clusters in sediment, water and WWTP samples. The Y-axis presents each of the ESBL genes, and the X-axis presents the number of ESBL-encoding plasmids. Each plasmid cluster is represented by a distinct color as indicated in the legend. Plasmid clusters occurring fewer than three times are grouped under “Other.”.

### Diversity of ESBL genes in *E. coli* isolates from water, sediment, and WWTP

Fourteen distinct ESBL genes were identified across sediment, water, and WWTP isolates, with seven genes shared among all sample types (**Fig 5, Table 1 in** **S4 File**). These common genes were also among the most prevalent across different regions (**Table 2 in** **S4 File**).

The most prevalent gene was *bla*_CTX-M-15_ (n = 331, 58%), detected widely across isolates from different regions and aquatic environments (**Table 3 in** **S4 File**). Other commonly detected genes included *bla*_CTX-M-1_ (n = 85, 15%)_,_ *bla*_CTX-M-14_ (n = 42, 7%), and *bla*_CTX-M-27_ (n = 41, 7%). WWTP influent samples exhibited the highest ESBL gene diversity. Some ESBL genes were restricted to a single region throughout almost the entire one-year sampling period: three genes in region A (*bla*_CTX-M-2_, *bla*_CTX-M-8,_ *bla*_CTX-M-9_) and one gene in region B (*bla*_CTX-M_).

Around 60% of isolates were predicted to carry plasmid-encoded ESBL genes, with *bla*_CTX-M-15_ linked to diverse plasmid clusters, while *bla*_CTX-M-1_ was mainly associated with a specific plasmid cluster (AA474) (**Fig 6**). Cluster AA474 included plasmids with replicon regions IncI-gamma/K1 (n = 94) and IncI1/B/O (n = 14).

### Comparison of sample types for detecting overall diversity of ESBL-producing *E. coli* in the aquatic environment

We assessed which sample type or combination of sample types best represents the overall diversity of STs, ESBL genes, and plasmid primary clusters of ESBL-producing *E. coli* in the aquatic environment. This evaluation was based on the proportions of detected characteristics (**Table 3**) and on significant differences in these proportions between sample types (**Table 1** in **S5 File**).

**Table 3:** Proportion of isolates from different sample types used to assess the overall ST, ESBL, and plasmid diversity of ESBL-producing E. coli in the environment.

Sample type	ST (n = 134)		ESBL (n = 14)		Plasmids (n = 60)	
	*n*	*%*	*n*	*%*	*n*	*%*
Sediment	63	47	9	64	34	57
Water	81	60	10	71	37	62
WWTP	57	43	12	86	32	53
Sediment + Water	110	82	14	100	60	100
Sediment + WWTP	95	71	14	100	60	100
WWTP + Water	108	81	14	100	60	100
All	134	100	14	100	60	100

ST diversity was most effectively captured in water samples, while ESBL genes and plasmid diversity were consistently detected across all individual sample types. No significant differences in any characteristics were observed between sediment and WWTP samples.

Combining water or sediment samples with other sample types significantly increased diversity across all characteristics compared to water or sediment samples alone. When WWTP samples were combined with other sample types, there was a significant increase in ST and plasmid diversity compared to WWTP samples alone, while no significant difference was observed in ESBL detection. Combining sample types enhanced the overall diversity detection. Combinations including water samples provided the greatest insight into ST diversity.

## Discussion

In this study, we characterized the diversity of ESBL-producing *E. coli* across multiple aquatic environments by analysing water, sediment, as well as WWTP influent samples. Prevalence varied among aquatic environments, yet overall occurrence was high, with clonal isolates identified even across geographically distant regions. Water samples captured the greatest diversity, while sediment and WWTP influents offered complementary insights into the overall diversity of ESBL-producing *E. coli* in the environment.

The WWTP influents exhibited the highest prevalence of ESBL-producing *E. coli* among all sample types, consistent with previous reports [31,32]. Water samples yielded more positives than sediment, likely reflecting ongoing or recent inputs into the environment [33,34]. Although fewer sediment samples were positive, those that were often contained higher bacterial loads, suggesting that sediment may act as a reservoir where bacteria accumulate and persist [34] (**Table 2 in** **S1 File**). The absence of positive sediment samples at sites with positive water samples could be explained by local hydrological and sediment characteristics, such as limited deposition or grain properties that hinder bacterial settlement and colonization [35].

Across aquatic environments, river sites located downstream of WWTP effluents showed the highest levels of ESBL-producing *E. coli,* followed by upstream sites and agricultural area. The elevated presence downstream compared with upstream sites suggests that WWTPs may represent an important source of ESBL-producing *E. coli* in this case. This is further supported by a higher prevalence of shared STs and clonal isolates between downstream sites and WWTP influent compared to downstream and upstream sites. Similar patterns have been reported in previous studies [36,37]. However, WWTPs are not the only potential contributors to riverine contamination; additional inputs may also increase microbial loads downstream [38,39]. Although agricultural impacts were not directly assessed in this study, river sites located in agricultural areas may reflect influences such as fertilization and field runoffs [40]. ESBL-producing *E. coli* was also detected in tributary sediment samples, likely linked to slower flow velocity, which facilitates bacterial settlement [41]. Other aquatic environments, including gravel pits, ponds, and natural lakes, exhibited low prevalence, likely due to the absence of direct discharges and limited wildlife-mediated transmission.

In terms of sample types, the highest ST diversity was observed in water from aquatic environments, compared with sediment and WWTP influents. The greater diversity in water is likely explained by the mixing of multiple sources and hydrological connectivity, while lower diversity in WWTP influents and sediment samples may result from the accumulation of persistent clones and stronger selective pressures [42].

Several STs showed region-specific prevalence; for example, ST16433 was dominant in region A and has been reported in humans and the environment [13]. ST12584 was prevalent in region B but has not yet been reported elsewhere, while ST5846 was dominant in region C and has been associated with animals in a Uruguayan study [43]. Across all regions and sample types, the most prevalent STs were ST131 (10.5%), ST38 (9.2%), ST10 (7.8%), ST69 (5.3%), and ST1193 (4.3%). These globally widespread, high-risk, human-associated pandemic lineages have been detected across humans, animals, and the environment. Their presence in all compartments suggests both a strong human influence on the environment and, conversely, a potential risk of transmission back to humans [44–47]. No seasonal variation in ST distribution was detected, consistent with previous studies [13,48].

Different sample types revealed similar representations of phylogroup prevalence, with A (27%), B1 (20%), B2 (24%), and D (20%) being the most common. Phylogroups A and B1 are considered less virulent and are usually predominant in the environmental sources, whereas phylogroups B2 and D, are more commonly associated with humans and often linked to pathogenic strains causing extraintestinal infections. The relatively balanced distribution observed in this study suggests that human-associated strains may be increasing in the environment [49,50].

Transmission patterns of ESBL-producing *E. coli* appear highly dynamic. Some clonal isolates were restricted to one or two sampling sites, while others were found across multiple sites, located also in two or three geographically distinct regions. Such patterns may reflect either a common source or transmission between sites, which is more likely to happen across distinct regions [51]. Clonal isolates occurred in various aquatic environments, most frequently in those under strong anthropogenic influence, such as downstream and upstream river sites, WWTP influents, and agricultural areas. Some clonal isolates persisted through the whole year, suggesting repeated inputs from contamination sources. Similar patterns have also been reported in other studies [31,52]. In our study, clonal isolates of ST2179 were consistently detected in both WWTP influents and the downstream site, indicating ongoing dissemination [36]. Clonal isolates were also found across different sample types. While overlap between nearby water and sediment samples is expected, clones found in both WWTP influent and environmental samples suggest potential transmission events. The dynamic transmission patterns in this study likely reflect combined influences of multiple factors, hydrological connectivity of river systems, wastewater inputs, and potential agricultural runoff at agricultural site within particular region. However, since rivers in the studied regions are not hydrologically connected, the presence of clonal isolates across distinct systems suggests additional dissemination routes, such as wildlife or human activities.

Most ESBL genes, including the predominant ones (*bla*_CTX-M-15,_ *bla*_CTX-M-1,_ *bla*_CTX-M-14,_ *bla*_CTX-M-27_), were detected across all sample types. Only a few genes were restricted to a single sample type, consistent with previous observations [53,54]. Many isolates carried ESBL genes on plasmids, which play an important role in antimicrobial resistance dissemination in the environment [55]. The most prevalent *bla*_CTX-M-15_ was encoded on multiple plasmid types, while *bla*_CTX-M-1_ was predominantly associated with specific plasmids, mostly with replicon region IncI-gamma/K1, which is consistent with previous findings [56,57].

The observation that water samples showed the highest ST diversity, while ESBL genes and plasmid types were detected across all sample types, can be explained by the differences in the entities being measured. STs represent the genetic background of individual bacterial strains, which tend to be highly diverse, particularly in environmental reservoirs such as water where continuous flow introduce new ST. In contrast, ESBL genes and plasmids can be shared horizontally across different strains and species. Consequently, while the number of unique STs is generally larger, the pool of ESBL genes and plasmid types is comparatively smaller and more broadly distributed, allowing their detection across all sample types.

One of the aims of this study was the comparison of how well do diverse sample types capture the diversity of genotypes, genes and plasmids. Water samples captured the highest ST diversity, while ESBL genes and plasmid diversity were generally detected across all sample types. Combining sample types improved the overall detection of diversity, particularly when water was paired with sediment or WWTP samples. Using a combination of sample types could help detect less common STs that might otherwise be missed, highlighting the potential benefit of strategic sampling strategies in environmental surveillance.

## Conclusion

This study detected a high overall prevalence of ESBL-producing *E. coli* across multiple aquatic environments in Slovenia. The transmission of ESBL-producing *E. coli* proved to be highly dynamic, with isolates able to persist over time, remaining localized to specific sites, or spreading across geographically distinct regions. Among all sample types, water provided the most comprehensive information on overall diversity, while adding sediment or WWTP samples provided complementary insights, offering a more complete picture of the diversity of ESBL-producing *E. coli* in the environment.

## Supporting information

S1 Fig Map of Slovenia showing the locations of sampling sites along three rivers. Each site is color coded by type of aquatic environment, as defined in the legend. The locations of nearby wastewater treatment plants (WWTP) effluents are also indicated. The map was generated in RStudio using open-access spatial data from the national Open Data portal of Slovenia (https://podatki.gov.si/pogoji-uporabe#). Sampling points were added approximately for schematic purposes.
(TIF)

S2 Fig Estimation of presumptive ESBL-producing *E. coli* colonies observed in sediment and water samples from each aquatic environment and WWTP influent samples. The chart presents the total number of presumptive ESBL-producing *E. coli* colonies observed on filter papers from sediment (orange) and water (blue) samples in each aquatic environment, and in WWTP influent samples (grey). The labels above (A, B, or C) indicate the region where each aquatic environment and WWTP influent were sampled. The numbers presented on the graph were calculated from countable plates only; numbers of uncountable plates (> 100 colonies per filter for water or sediment samples, and > 250 colonies per plate for WWTP samples) are summarized separately.
(TIF)

S3 Fig Number of ESBL-producing *E. coli* isolates included in the analysis. The chart shows the total number of ESBL-producing *E. coli* isolates found in sediment (orange) and water (blue) in each aquatic environment, as well in WWTP influent samples (grey). The labels above (A, B, in C) indicate the region where each aquatic environment and WWTP influent were sampled. Numbers below the bars indicate the number of positive samples (in which ESBL-producing *E. coli* was confirmed) out of the total number of samples collected.
(TIF)

S4 Fig STs found in each sampling month and in each region. The figure shows the distribution of all STs detected in each sampling time point and region, across all three different sample types: sediment, water, and WWTP influent samples.
(TIF)

S5 Fig Presence of clonal groups across sampling time points in each region. The figure shows the distribution of clonal groups in each sampling month and region, across all three different sample types: WWTP, water, and sediment. Each MST clonal group is represented by a distinct color and labeled with a ‘C’ followed by a number (e.g., C6). The number after the underscore indicates ST of the isolates within that clonal group (e.g., C6_44 represents clonal group 6 containing ST44 isolates).
(TIF)

S6 Fig Distribution of *E. coli* clonal isolates in river sites upstream and downstream of WWTP effluent, and in WWTP influents. The figure shows identified clonal groups in river sites upstream and downstream of WWTP effluents, and in WWTP influents, in each region separately. Shared clonal isolates between upstream and downstream sites and between downstream site and WWTP influent, are connected by lines, indicating the potential dissemination and contribution of WWTP to downstream river area of WWTP effluent. Each line color represents a distinct MST clonal group, which are labeled with “C” as explained in Figure S5. Sampling locations are indicated below each sampling site.
(TIF)

S1 File Table 1: Description of sampling sites and number of positive samples in each site. Table 2: Number of positive samples and presumptive ESBL-E. coli, calculations of CFUs and final number of confirmed ESBL-*E. coli.* Table 3: Metadata for all 315 collected samples. Table 4: Metadata for all 564 sequenced ESBL-producing *E. coli* isolates. Table 5.1: Significance of differences in MLST-STs diversity between sample types. Table 5.2: Significance of differences in MLST-STs diversity between regions (A) and differences of ST composition between regions (B) Table 5.3: Significance of differences in MLST-STs diversity between aquatic environments. Table 5.4: Significance of differences in phylogroups diversity between sample types based on richness (A) and differences of phylogroups composition between sample types (B) Table 5.5: Significance of differences in phylogroups diversity between regions based on richness (A) and Shannon index (B) and differences of phylogroups composition between regions (C) Table 6: Prevalence of phylogroups in all three sample types: sediment, water and WWTP. Table 7: ENA submission data for each isolate.
(XLSX)

S2 File Table 1: Distribution of ST in all three sample types: sediment, water and WWTP influents. Table 2: Distribution of ST in each region. Table 3: Distribution of ST in all three sample types in each region. Table 4: Distribution of ST in each aquatic environment and WWTP influents. Table 5: Distribution of ST in different sampling time points. Table 6: Distribution of ST in different sampling time points in each region.
(XLSX)

S3 File Table 1: MST clonal groups found in each sample type: sediment, water and WWTP. Table 2: MST clonal groups found in different regions. Table 3: MST clonal groups found in different aquatic environments and WWTP influent samples. Table 4: MST clonal groups found in different sampling time points. Table 5: MST clonal groups found in all sampling site across three different regions. Table 6: MST clonal groups found in each sample type in each region. Table 7: MST clonal groups found in different aquatic environments and WWTP influent samples in each region. Table 8: MST clonal groups found in each sampling time point in each region.
(XLSX)

S4 File Table 1: Distribution of ESBL genes across different sample types: sediment, water and WWTP influents. Table 2: Distribution of ESBL genes across different sample types in each region. Table 3: Distribution of ESBL genes across different aquatic environments and WWTP influent.
(XLSX)

S5 File Table 1: Significance of differences in the ability of different sample types and their combinations to detect overall ST, ESBL, and plasmid diversity of ESBL-producing *E. coli.* (XLSX)
